# Robotic-Assisted Laparoscopic Transabdominal Preperitoneal Repair of Littre’s Hernia: A Case Presentation and Literature Review

**DOI:** 10.7759/cureus.92850

**Published:** 2025-09-21

**Authors:** Antonio Melhem, Rollin William Johnson, Mina Iskaros, Andrew Godwin, David Buchin

**Affiliations:** 1 General Surgery, Wyckoff Heights Medical Center, New York, USA; 2 Vascular Surgery, Englewood Health, Englewood, USA; 3 General Surgery, Northwell Health, Huntington, USA

**Keywords:** incarcerated inguinal hernia, littre’s hernia, meckel’s diverticulum, robotic hernia repair, robotic surgical procedures

## Abstract

Meckel’s diverticulum is a common gastrointestinal anomaly that can remain unnoticed until adulthood and may present in a hernia sac, known as Littre’s Hernia. Littre’s hernia is a very rare entity in acute care surgery, which can be missed during index surgery or during the initial surgical evaluation. We present the case of an incarcerated Littre’s hernia, found intraoperatively during a robotic-assisted laparoscopic hernia repair, in an 82-year-old female patient. Based on intraoperative findings, a simple diverticulectomy was performed, and a mesh was used to reinforce the repair of the hernia. In addition, we are presenting a review of the current literature on Meckel’s diverticulum and Littre’s hernia, particularly among adult patients.

## Introduction

Meckel’s diverticulum (MD) is one of the most common congenital anomalies of the gastrointestinal tract and is caused by incomplete obliteration of the omphalomesenteric duct during embryologic development [1]. In most cases, MD is characterized by the presence of two different types of ectopic tissues: gastric and pancreatic. The presence of ectopic gastric tissue results in acid secretion, causing gastrointestinal bleeding. Other common presentations include intussusception, small bowel obstruction, diverticulitis, and, rarely, as a hernia, also known as Littre’s hernia. MD can be found in the hernia sac of an inguinal, femoral, obturator, spigelian, ventral, or even incisional hernia. Littre’s hernia can be found in up to 1% of inguinal hernias among children and is rarely seen among adults, which makes its clinical relevance both interesting and challenging to acute care surgeons when it comes to diagnosis and management. Many clinicians use the rule of 2s when suspecting a MD in their diagnosis. The incidence of MD is around 2% of the population; only 2% show symptoms; it usually affects those who are two years and younger; males are twice as prone to this pathology; it is found at 2 feet from the ileocecal valve; it measures 2 inches in length; and it has two types of mucosal lining [2,3]. A 2019 systematic review reported 53 patients presenting with a Littre’s hernia, with most being incarcerated [4]. In this manuscript, we describe the case of an 82-year-old female patient who presented with an incarcerated right-sided inguinal hernia and was found to have an MD within it during the operative repair. This case report has been reported in line with the SCARE checklist.

## Case presentation

**Figure 1:**
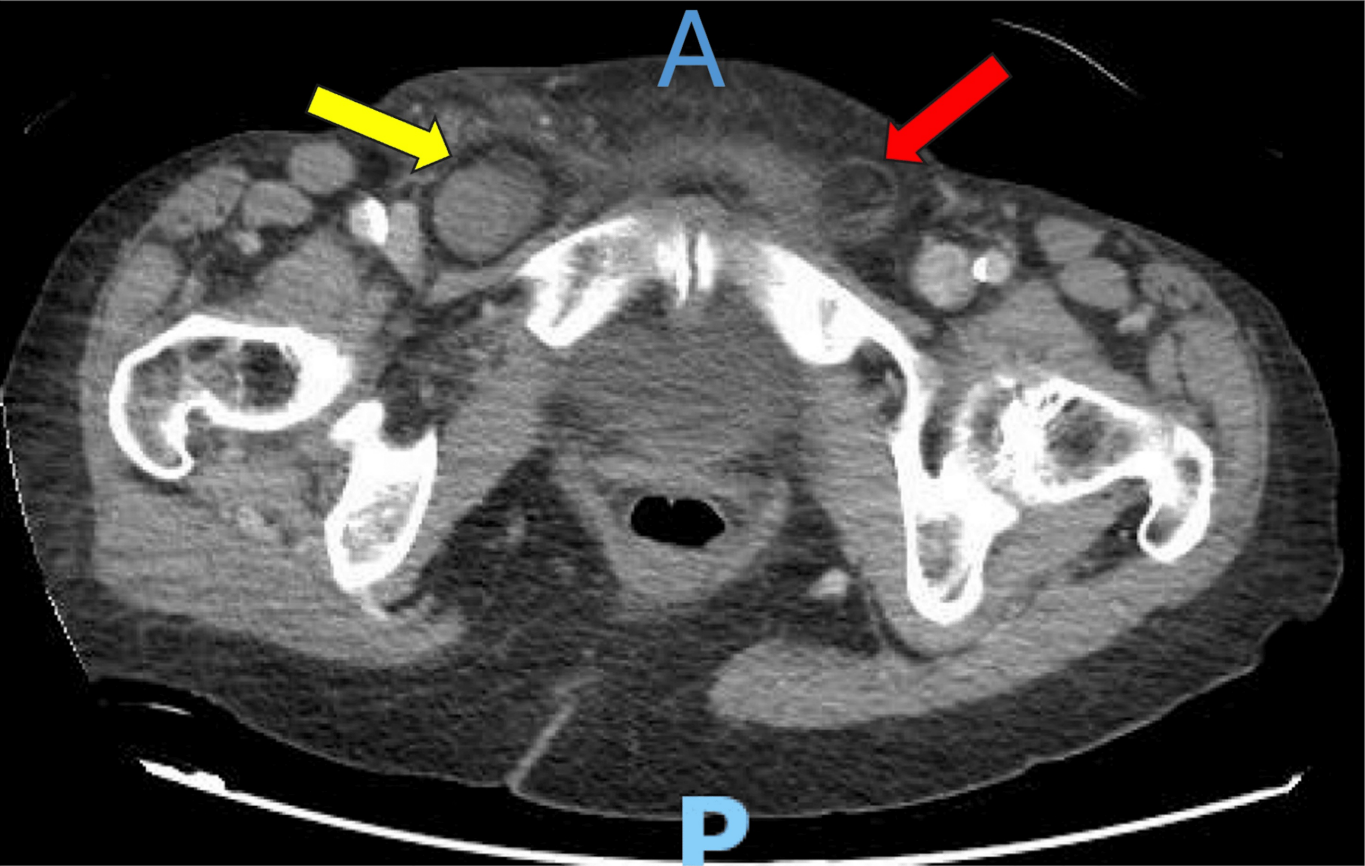
Axial view of the CT of the pelvis with intravenous contrast A is the anterior side; P is the posterior side. The yellow arrow is pointing at the bowel-containing right-sided inguinal hernia. The red arrow is pointing at the fat-containing left-sided inguinal hernia.

An 82-year-old female presented in June 2025 to our emergency department with a same-day history of an acutely painful bulge at the level of her right groin. The patient stated that a few years ago, she had an open repair of a left-sided inguinal hernia with a mesh placed during the procedure. The patient was also complaining about nausea and a one-time emesis episode. She denied any constipation or obstipation. She never noticed this bulge before this admission. During our examination, the patient was in discomfort, but her vitals were within normal range. There was a bulging mass in the right inguinal area, firm, tender to palpation, and non-reducible, despite applying icepacks and placing the patient in the Trendelenburg position. The overlying skin did not show any changes or abnormalities, and her abdominal examination was benign. A decision was made to take the patient to the operating room for inguinal hernia repair. A CT of the abdomen and pelvis with intravenous and oral contrast was obtained before attempting any reduction at bedside, which showed a right inguinal hernia containing a short segment of small bowel with no evidence of bowel obstruction or ischemia; a left-sided fat-containing hernia was also noted (Figure 1).

Given that the patient did not show any obstructive symptoms, radiographic evidence not concerning for compromised bowel, a benign abdominal examination, and bilateral inguinal hernias were seen on CT, we opted to adopt a robotic-assisted laparoscopic transabdominal preperitoneal approach. A large direct incarcerated hernia was identified on the right side, and upon dissection of the cord structures, an MD was identified. The remainder of the abdomen was inspected, and the bowels were unremarkable. The base of the MD was approximately greater than 2 cm with a long body, warranting a simple diverticulectomy via laparoscopic Endo GIA stapler. A synthetic mesh was used to reinforce the repair. A small left-sided, fat-containing hernia was identified as well, and a decision was made to proceed with primary repair and mesh placement. At the end of the case, the patient was extubated and had an uneventful postoperative course, with diet advanced as tolerated and being stable for discharge on postoperative day one. The pathology report showed that the mucosa of the specimen was not viable, with spots of transmural inflammation. No ectopic gastric mucosa was found in the specimen. The patient presented for follow-up at our clinic after two weeks. The surgical sites were inspected and were deemed to be healing well. The inguinal regions were also examined, and no signs of recurrence, serum, or infection were noted. The pathology report was reviewed with the patient. Full insight and details about the pathology were disclosed to the patient. Moreover, we reviewed with the patient her postoperative instructions to help reduce the risk of recurrence, infection, or other common complications.

## Discussion

Littre’s hernia is an infrequent clinical presentation of MD. It was first described by Alexis de Littre (1700) in a three-patient case series [5]. The incidence of Littre’s hernia remains uncertain, but a few papers reported that up to 1% of patients with MD will develop Littre’s hernia in their lifetime [6,7]. Surgeons must carefully distinguish an MD from a Richter hernia, where the antimesenteric portion of the bowel herniates, but no MD is found, as listed in the literature [8].

Littre’s hernia was once categorized into two different subtypes: a true hernia, which is more frequent and contains only an MD; and a mixed hernia, which encompasses a segment of the small intestine along with the MD [9]. The latter is less commonly reported in the literature. Our case describes a mixed type, where the bowel was completely preserved and intact. The pathology report indicated that our specimen lacked any viable mucosa, and there were areas with transmural inflammation. According to the literature, an MD shows a relative resistance to ischemia when compared to a small bowel strangulated hernia [10,11]. Our pathology report findings align with the hypothesis proposed by Mirza (2007) and Horkoff et al. (2014) that a strangulated MD is more resistant to ischemia compared to the small bowels, mainly due to either lacking a full muscular layer or containing rigid ectopic tissues, which may slowly necrose without triggering an alarming systemic and inflammatory response [10,11]. This highlights the challenging diagnostic nature of a Littre’s hernia and the importance of keeping in mind a high index of suspicion of when to intervene, mainly in the absence of signs of peritonitis. Moreover, a clinical distinction between a Littre’s hernia and a herniated small bowel loop is almost impossible, and the diagnosis is usually made intraoperatively.

The diagnosis of an inguinal hernia is usually clinical, based on history and physical examination. If not clear, an ultrasound or a CT scan might be ordered. In some instances, a CT scan is necessary if clinical examination is equivocal about bowel viability, which dictates the timing of surgical repair. In most cases, a definitive diagnosis of Littre’s hernia is challenging to make from CT alone and is made from intraoperative findings. A CT scan provides valuable information and can help differentiate an inguinal hernia from a femoral and an obturator hernia, in addition to providing information about bowel viability and intraperitoneal contamination.

The surgical options for a Littre’s hernia include resection for immediate symptom relief, a high risk of symptomatic recurrence if an MD is left behind, and potential complications such as bleeding, especially if ectopic gastric mucosa is found within it [12]. MD resection can be via a simple diverticulectomy or a segmental small bowel resection. The decision is mostly made based on its size and the size of its base. When an MD is long with a narrow base, a simple diverticulectomy is enough, but when it is short with a broad base, a segmental small bowel resection is preferable. In theory, chances of having the ectopic tissue spread to the small bowel are higher with a short MD compared to a long one; thus, a segmental resection reduces the risk of future bleeding [13]. The decision to use a mesh and, subsequently, the appropriate mesh type, is made intraoperatively based on the hernia size, bowel viability, and field contamination. If the inspection and appropriate resection are not possible through the laparoscopic incisions or the open inguinal approach, then a midline laparotomy is needed.

Robotic-assisted surgical procedures have become more common, especially when working on inguinal, femoral, or obturator hernias. The proficient use of a robot provides multiple advantages, including minimal postoperative pain, early return to normal activity compared to open cases, and, more importantly, an improved field of visualization, finer dissection, and enhanced dexterity when compared to laparoscopic cases. Moreover, in a patient with a history of an open hernia repair and now presenting with a recurrence, a robotic approach allows the surgeon to work in a different tissue field rather than in the same, previously used, and now scarred surgical field. Further, a robotic approach allows for bilateral inguinal hernia repair, similar to our case, using the same three 5 mm skin incisions, rather than creating two separate and long supra-inguinal incisions, which could increase the risk of infection and recurrence. Our decision to use the robot was based on a previous history of open hernia repair, the presence of bilateral hernias on imaging, and the technical advantages provided by this novel approach.

The largest paper about Littre’s hernia, to date, is a systematic review which included 53 cases, with femoral hernias being the most common (39.6%) followed by inguinal hernias (34%) [4]. In their literature review, Schizas et al. (2019) showed that 45% of Littre’s hernia cases were strangulated and up to 9% proceeded to bowel perforation, while 34% had associated bowel obstruction. They also noted a mortality rate of 2% resulting from multi-organ failure following a perforated Littre’s hernia [4]. Although more men are affected with MD, the incidence of Littre’s hernia is more frequent among women (60.4%), mostly related to their higher incidence of femoral and obturator hernias [4]. Our patient, an elderly female, aligns with this demographic trend. Littre’s hernia remains a rare entity, and clinicians must keep it in their list of differential diagnoses. Given the slow progress of ischemic MD in a hernia without showing overt symptoms, surgeons must have a low threshold to take the patient to the operating room when they have high suspicions.

Finally, our patient’s findings did not align with the classical rule of 2s for MD. Our patient was an elderly female, while the rule of 2s states that MD is more common among men and those who are less than two years of age. Moreover, a classic MD typically has two types of ectopic tissues, while the one in our case did not have any. To note, our patient’s presenting white cell count and C-reactive protein count were normal preoperatively, which is in line with the absence of bowel obstruction and overt signs of systemic toxicity. Given the uneventful recovery period before discharge, no labs were drawn after surgery, and the patient was discharged on postoperative day one.

## Conclusions

Littre’s hernia is a rare entity; the diagnosis can be challenging outside the operating room, but the management is similar to any bowel-containing hernia. We presented a case of a mixed Littré’s inguinal hernia involving a segment of viable small bowel and an MD, which was found to be necrotic. The patient safely underwent a robotic-assisted laparoscopic hernia repair with mesh placement and was discharged one day after the surgery. The goal of this paper is to highlight the importance of promptly recognizing and managing a bowel containing inguinal hernia amongst adults, regardless of the presence of obstructive and/or systemic symptoms. Surgeons should keep a low threshold for possibly dealing with a Littre's hernia, or a Richter hernia, which can present with minimal to no symptoms, and can inadvertently develop into a case of bowel compromise and an acute abdomen.

## References

[1] Sagar J, Kumar V, Shah DK (2006). Meckel's diverticulum: a systematic review. J R Soc Med.

[2] Hansen CC, Søreide K (2018). Systematic review of epidemiology, presentation, and management of Meckel's diverticulum in the 21st century. Medicine (Baltimore).

[3] Francis A, Kantarovich D, Khoshnam N, Alazraki AL, Patel B, Shehata BM (2016). Pediatric Meckel's diverticulum: report of 208 cases and review of the literature. Fetal Pediatr Pathol.

[4] Schizas D, Katsaros I, Tsapralis D (2019). Littre's hernia: a systematic review of the literature. Hernia.

[5] Littre A (2025). Littre A. Observation sur une nouvelle espèce de hernie. https://hal.science/ads-00104348/document.

[6] Ioannidis A, Karanikas I, Koutserimpas C, Velimezis G (2018). Combined Littre and Richter's femoral hernia: an extremely rare intra-operative finding. G Chir.

[7] Racy M, Ramesh S (2013). Littré meets de Garengeot: Meckel's diverticulum and appendix in a femoral hernia. Ann R Coll Surg Engl.

[8] Weinstein BM (1938). Strangulated Littre’s femoral hernia with spontaneous fecal fistula: case report with a review of the literature. Ann Surg.

[9] López-Lizárraga CR, Sánchez-Muñoz MP, Juárez-López GE, Pelayo-Orozco L, De la Cerda-Trujillo LF, Ploneda-Valencia CF (2017). A rare case of a strangulated Littre's hernia with Meckel's diverticulum duplication. Case report and literature review. Int J Surg Case Rep.

[10] Mirza MS (2007). Incarcerated Littre's femoral hernia: case report and review of the literature. J Ayub Med Coll Abbottabad.

[11] Horkoff MJ, Smyth NG, Hunter JM (2014). A large incarcerated Meckel's diverticulum in an inguinal hernia. Int J Surg Case Rep.

[12] Dunn TM, Markgraf WH (1962). Littre hernia--incarcerated Meckel's diverticulum. Am J Surg.

[13] Blouhos K, Boulas KA, Tsalis K (2018). Meckel's diverticulum in adults: surgical concerns. Front Surg.

